# Supplementary material for: Structure-based characterization of novel TRPV5 inhibitors
Source: eLife. 2019 Oct 25;8:e49572. doi: 10.7554/eLife.49572 (PMC6834369; doi:10.7554/eLife.49572)
Supplement: Figure 1—source data 1. — ZINC IDs and 2D chemical structures for the 65 unique chemical scaffolds identified in the in silico compound screen. [file elife-49572-fig1-data1.pdf]

Figure 1-source data 1

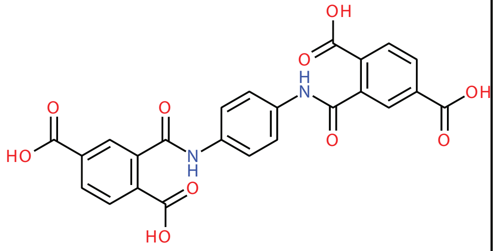
ZINC000002066182

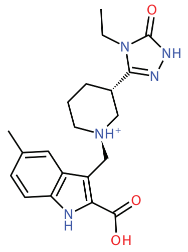
ZINC000067871925

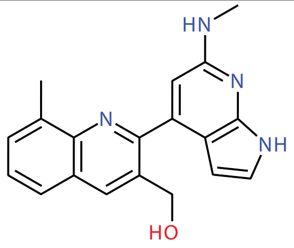
ZINC000072168826

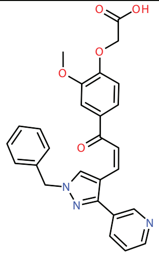
ZINC000096305150

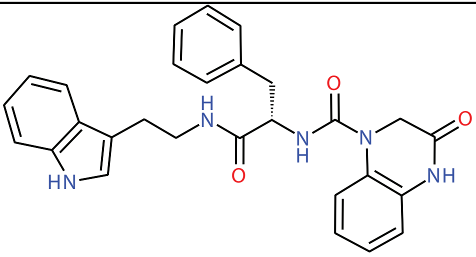
ZINC000008764705

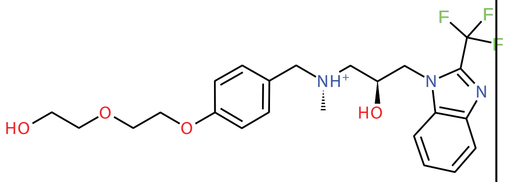
ZINC000257316788

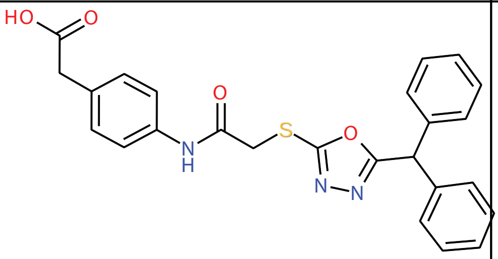
ZINC000009200134

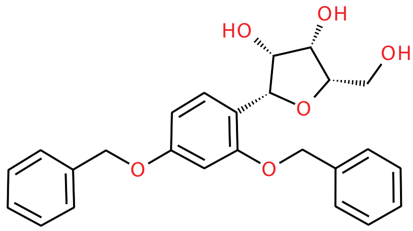
ZINC000005385249

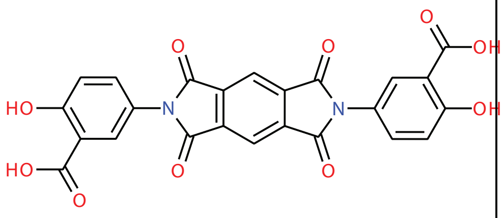
ZINC000003143326

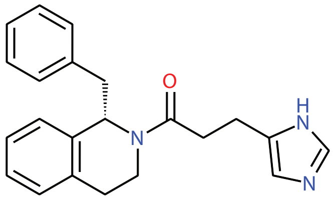
ZINC000189545218

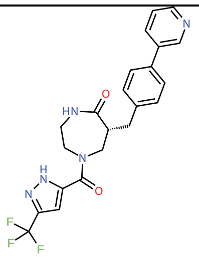
ZINC000019340733

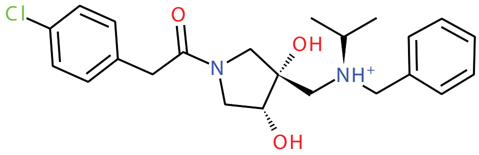
ZINC000585290561

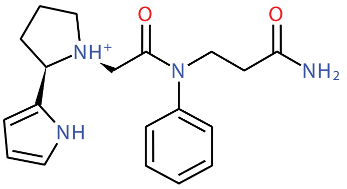
ZINC000048287288

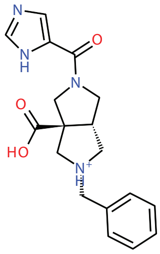
ZINC000065502652

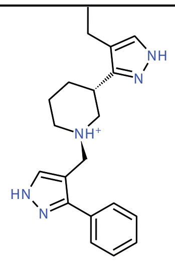
ZINC000077564673

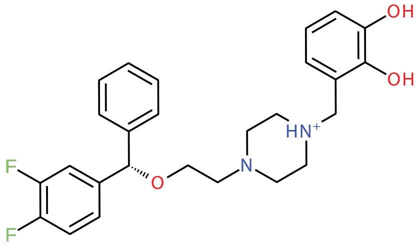
ZINC000023326129

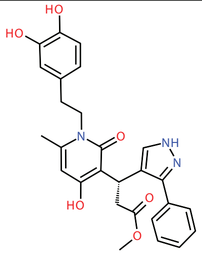
ZINC000253400660

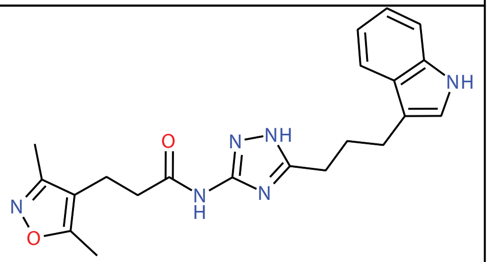
ZINC000079490144

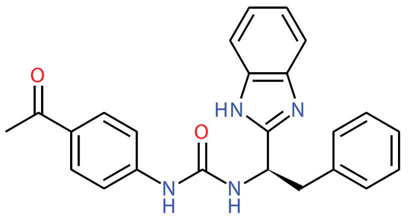

ZINC000040138964

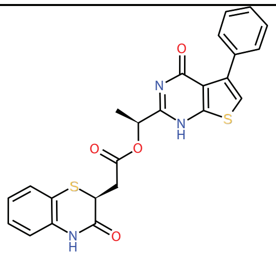

ZINC000009155420

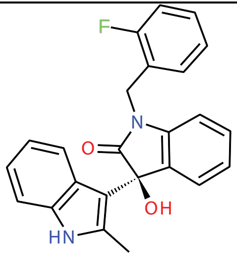

ZINC000004842852

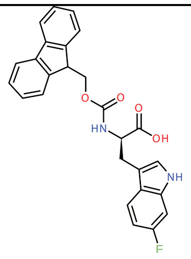

ZINC000040566535

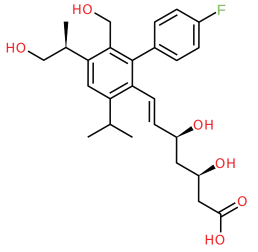

ZINC000065739947

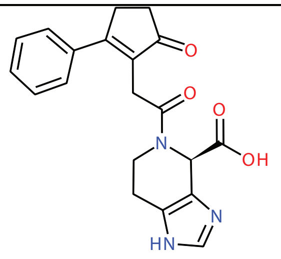

ZINC000072407626

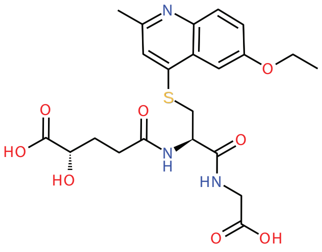

ZINC000009344575

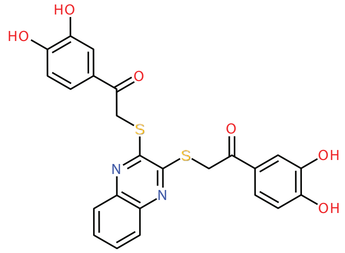

ZINC000334162937

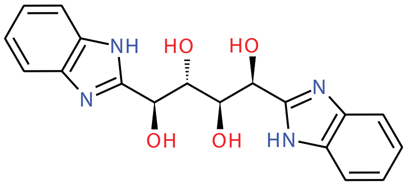

ZINC000018254317

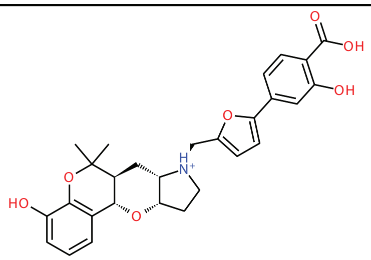

ZINC000257283810

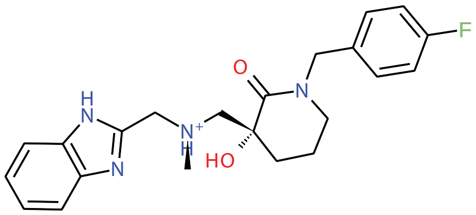

ZINC000053523490

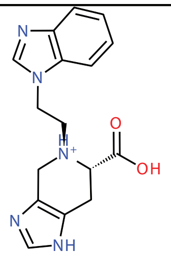

ZINC000097393918

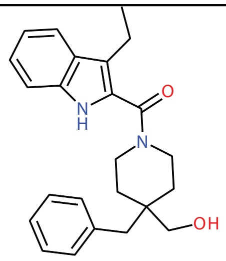

ZINC000067869160

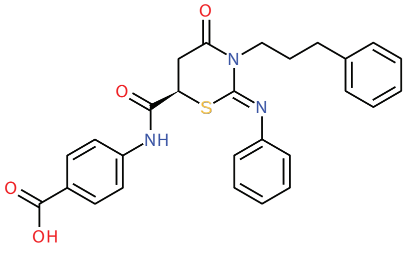

ZINC000012629272

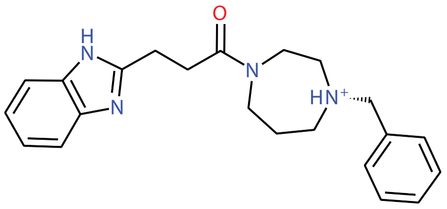

ZINC000029099048

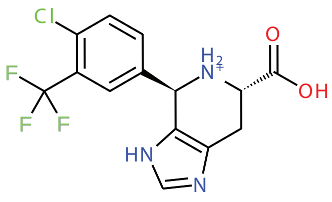

ZINC000005067315

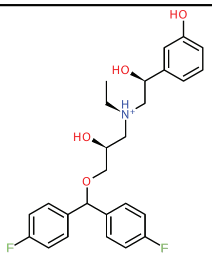

ZINC000012541229

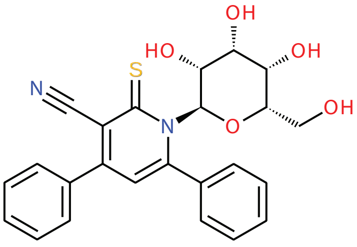

ZINC000006004557

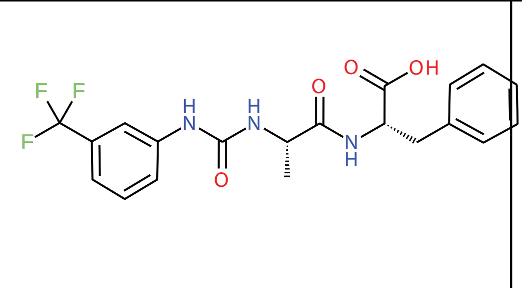

ZINC000016322280

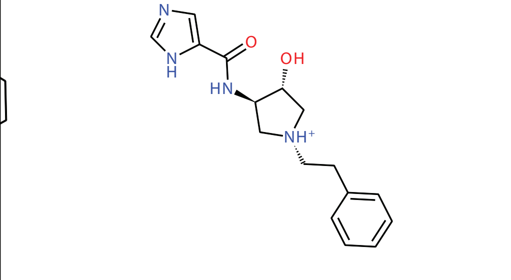

ZINC000257264474

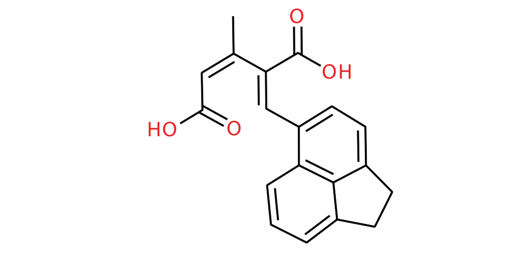

ZINC000002293820

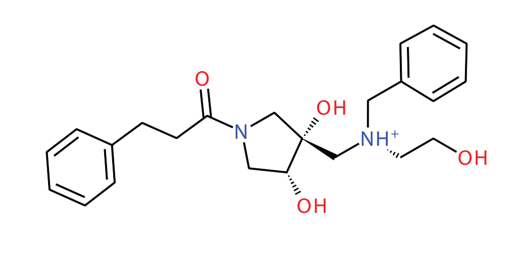

ZINC000585292138

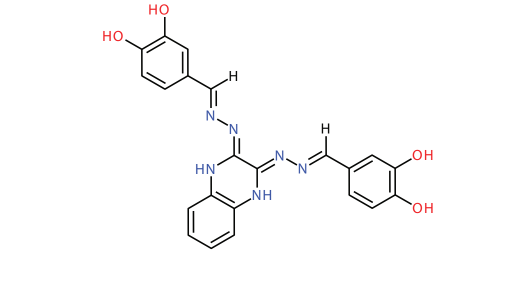

ZINC000252635502

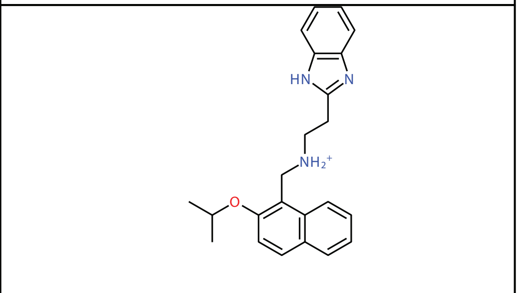

ZINC000113401229

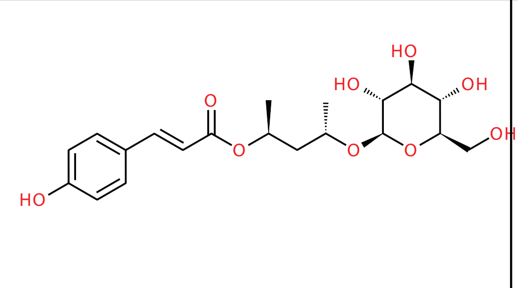

ZINC000031168775

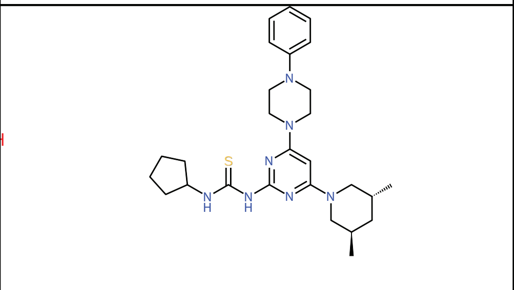

ZINC000226006254

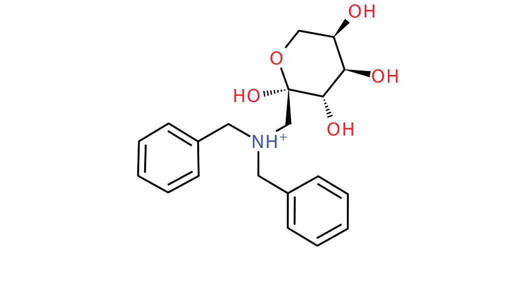

ZINC000004350985

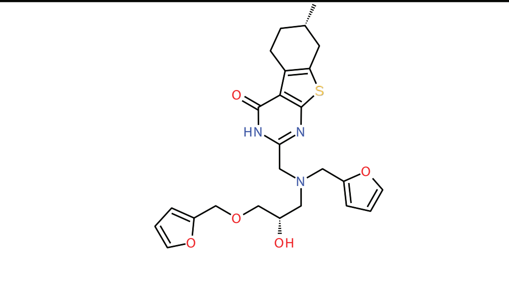

ZINC000036208021

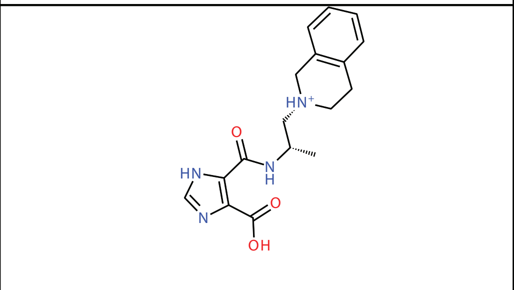

ZINC000299768831

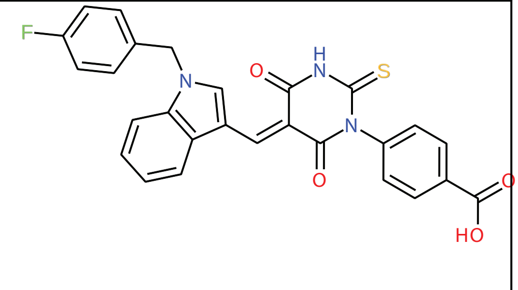

ZINC000409195867

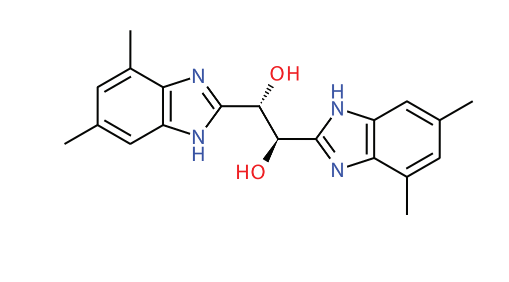

ZINC000255189665

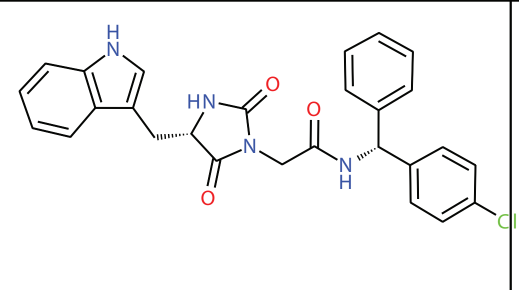

ZINC000009514316

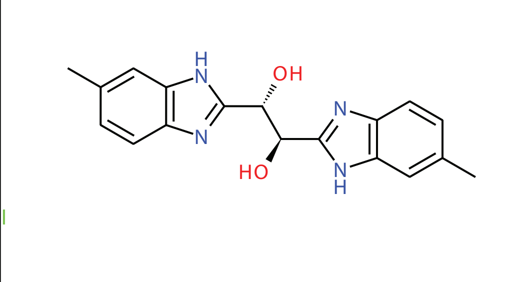

ZINC000013116430

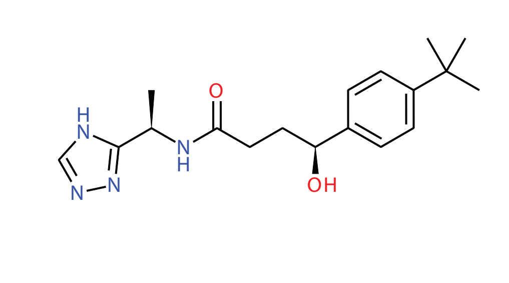

ZINC000281297333

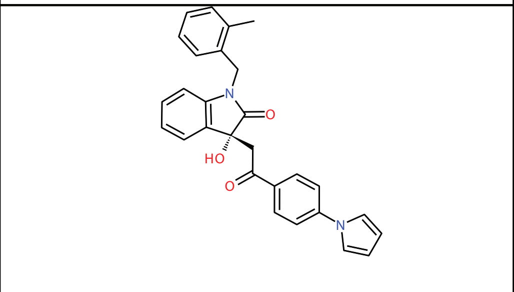

ZINC000004123130

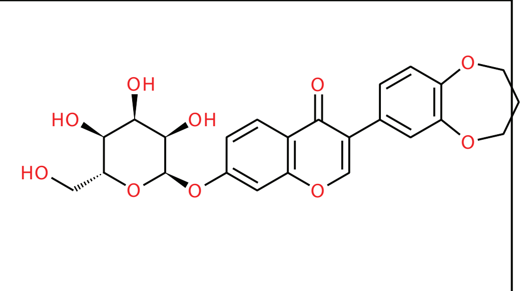

ZINC000105365609

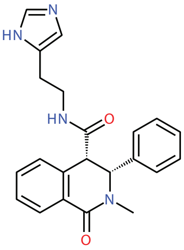

ZINC000036358455

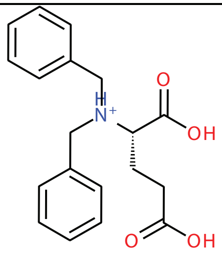

ZINC000002028241

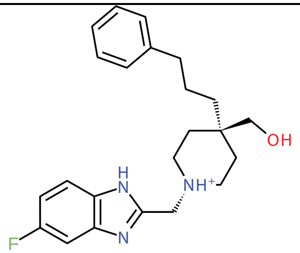

ZINC000011664563

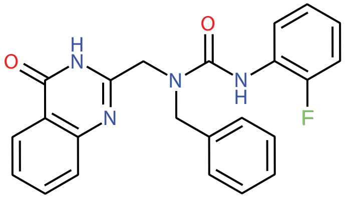

ZINC000005626366

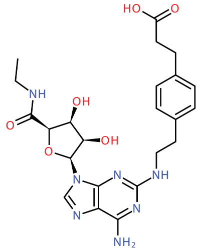

ZINC000009228229

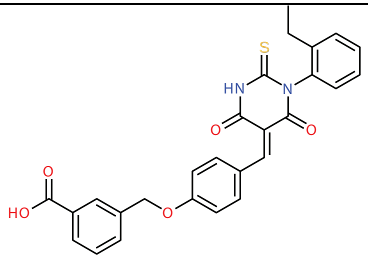

ZINC000408780943

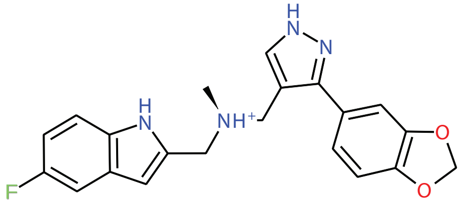

ZINC000019147573

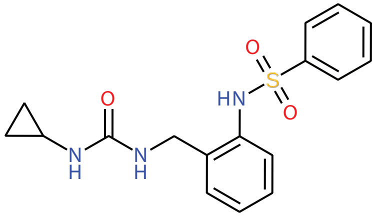

ZINC000065589127

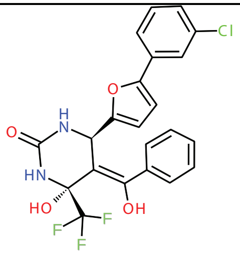

ZINC000002459082

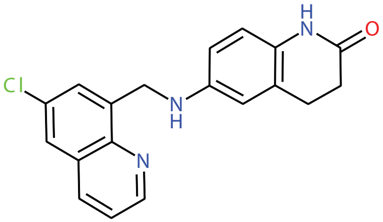

ZINC000095428543

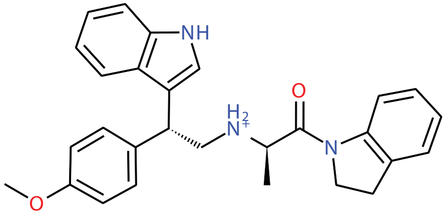

ZINC000009579411
