# Supplementary material for: Structure-based characterization of novel TRPV5 inhibitors
Source: eLife. 2019 Oct 25;8:e49572. doi: 10.7554/eLife.49572 (PMC6834369; doi:10.7554/eLife.49572)
Supplement: Figure 1—source data 2. — Listed are compounds identified in the SBVS that showed no effect on rbTRPV5 current in our system. The effect of each compound was tested at the listed concentration. N indicates the number of replicates tested. [file elife-49572-fig1-data2.docx]

**Figure 1-source data 2**

| **Number** | **ZINC ID** | **Other name(s)** | **N** | **Concentration** |
| --- | --- | --- | --- | --- |
| 1 | ZINC04842852 |  | 2 | 10 µM |
| 2 | ZINC11664563 |  | 2 | 10 µM |
| 3 | ZINC19147573 |  | 2 | 10 µM |
| 4 | ZINC53523490 |  | 2 | 10 µM |
| 5 | ZINC36358455 |  | 1 | 10 µM |
| 6 | ZINC65502652 |  | 1 | 10 µM |
| 7 | ZINC67871925 |  | 1 | 10 µM |
| 8 | ZINC72168826 |  | 1 | 10 µM |
| 9 | ZINC72407626 |  | 5 | 10 µM |
| 10 | ZINC77564673 |  | 4 | 10 µM |
| 11 | ZINC299768831 |  | 3 | 10 µM |
| 12 | ZINC40566535 |  | 2 | 10 µM |
| 13 | ZINC9579411 |  | 2 | 10 µM |
| 14 | ZINC9514316 |  | 2 | 10 µM |
| 15 | ZINC12541229 |  | 2 | 10 µM |
| 16 | ZINC48287288 |  | 1 | 10 µM |
| 17 | ZINC29099048 |  | 1 | 10 µM |
| 18 | ZINC65589127 |  | 1 | 10 µM |
| 19 | ZINC95428543 |  | 1 | 10 µM |
| 20 | ZINC189545218 |  | 1 | 10 µM |
| 21 | ZINC12629272 |  | 1 | 3 µM |
| 22 | ZINC02066182 |  | 1 | 3 µM |
| 23 | ZINC03143326 |  |  | did not dissolve |
| 24 | ZINC79490144 |  | 1 | 3 µM |
| 25 | ZINC09344575 |  |  | did not dissolve |
| 26 | ZINC334162937 |  | 3 | 3 µM |
| 27 | ZINC02293820 |  |  | did not dissolve |
| 28 | ZINC04123130 |  | 3 | 3 µM |
| 29 | ZINC36208021 | V016-9987 | 1 | 3 µM |
| 30 | ZINC31168775 | NP-014292 | 1 | 3 µM |
| 31 | ZINC16322280 | LT00723737 | 1 | 3 µM |
| 32 | ZINC05067315 | EB12699 | 2 | 3 µM |
| 33 | ZINC40138964 | PB447083392 | 1 | 3 µM |
| 34 | ZINC23326129 | V014-6317 | 1 | 3 µM |
| 35 | ZINC08764705 | PHAR199755 | 3 | 3 µM |
| 36 | ZINC257316788 |  | 3 | 3 µM |
| 37 | ZINC19340733 |  | 5 | 3 µM |
| 38 | ZINC585290561 |  | 8 | 3 µM |
| 39 | ZINC257283810 |  | 2 | 3 µM |
| 40 | ZINC257264474 |  | 3 | 3 µM |
| 41 | ZINC281297333 |  | 1 | 3 µM |
