# Supplementary material for: Structure-based characterization of novel TRPV5 inhibitors
Source: eLife. 2019 Oct 25;8:e49572. doi: 10.7554/eLife.49572 (PMC6834369; doi:10.7554/eLife.49572)
Supplement: Figure 1—source data 3. — ZINC IDs and 2D chemical structures for the ZINC9155420 derivatives that did not alter TRPV5 activity. The effect of each compound was tested at the listed concentration. N indicates the number of replicates tested. [file elife-49572-fig1-data3.docx]

**Figure 1-source data 3**

| **2D Structure** | **ZINC ID** | **Other name (s)** | **N** | **Concentration** |
| --- | --- | --- | --- | --- |
| 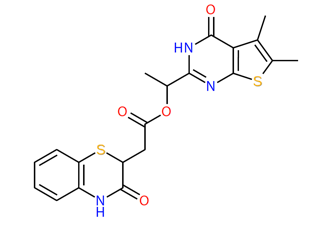 | ZINC09389941 | ZINC09389950 | 2 | 10 µM |
| 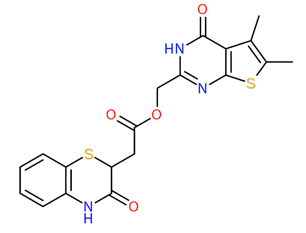 | ZINC25054165 | Z54077885, ZINC25054160 | 2 | 10 µM |
| 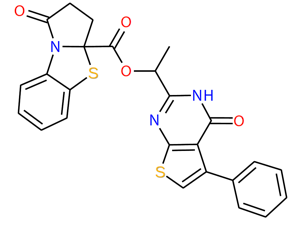 | ZINC09960474 | ZINC09960477 | 1 | 10 µM |
| 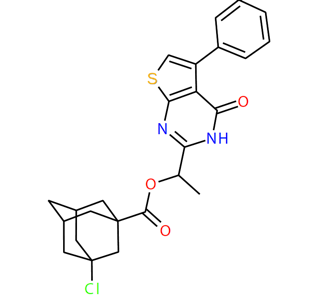 | ZINC13148573 | ZINC13148574 | 2 | 10 µM |
